# Modelling and Experimental Study of Moisture Transport in Wood, Taking into Account Diffusion and the Accompanying Adsorption of Water Vapour by Cell Walls

**DOI:** 10.3390/ma14010017

**Published:** 2020-12-22

**Authors:** Andrzej Kucharczyk, Kamil Pawlik

**Affiliations:** Civil Engineering and Architecture Faculty, Opole University of Technology, 45-061 Opole, Poland; k.pawlik@po.edu.pl

**Keywords:** water vapour, bound water, diffusion, adsorption, wood, sorption experiment

## Abstract

The paper presents a model of moisture transport in wood taking into account diffusion and the accompanying adsorption of water vapour through the skeleton. A two-parameter form of the source term was proposed, depending on the distance of the current moisture content (MC) from the equilibrium state. The tests on cubic samples with a side of 2 cm were carried out which allowed to determine the coefficients of the proposed model on the basis of the reverse method. The tests were performed for pine, larch, oak and ash in all directions of orthotropy. Tests on thin samples were also performed to verify the source term.

## 1. Introduction

Wood is widely used in construction as a structural and finishing material. The properties of wood such as strength, dimensional stability and durability are strongly dependent on its humidity. This means that the spatial distribution of moisture and its changes versus time play a key role in predicting its behaviour. The most important from the point of view of material engineering is the transport of moisture in the range of the moisture content of the wood corresponding to dry conditions and the moisture content occurring at full saturation of the fibres, i.e., in the moisture content from 0% to 30% [1,2]. In this range of moisture in wood there is water in the form of water vapour and water bound by surface forces, and changes in its content lead to shrinkage or swelling of the wood [3].

One of the first models for predicting moisture distributions in the hygroscopic range under isothermal conditions are based on the balance of total moisture diffusion. These models, although they do not fully explain the physical problem and the results obtained by means of the diffusion equation cannot always be matched to experimental data, which was analysed by Shi [4], are attractive due to the small number of parameters needed to describe the phenomenon. On the other hand, if the diffusion coefficient is assumed to depend on the moisture content, a very good fit of the model to experimental data is obtained [5,6].

Recently, more advanced models have also been developed, which analyse the combined flow of vapour and bound water [7,8,9,10,11,12]. These models assume that vapour and bound water are transported by diffusion. However, the form of the diffusion coefficient is not entirely clear. Siau [13] assumed that the resistance to vapour flow is influenced by both cell lumens and cell walls. Therefore, the coefficient can only be considered as an apparent diffusion coefficient. In fact, it is assumed that the vapour passes through the cell walls. On the one hand, it is adsorbed on the cell wall and then desorbed on the other. In this case, the diffusion coefficient should depend on the moisture content, e.g., in [14]. However, this is only one theory of the mechanism of vapour transmission. According to Dinwoodie [15], the main vapour transport route leads through pits in cell walls. A diffusion coefficient independent of moisture content is then to be expected. This case was assumed and confirmed by the following analyses.

The issue of the transport of bound water itself is also not fully explained. According to Kärger and Valiullin [16] diffusion in mesopores is the result of the interplay of several mechanisms. Thus, at partial pore-space saturation one may distinguish between surface diffusion and the process of mass transfer through the pore gas phase. On the other hand Hozjan and Svensson [11] claim that the diffusion of bound water in the porous structure is very slow compared to the diffusion of vapour. Therefore, for moisture transport in larger wood samples, the transport of bound water is minor importance and may be omitted [11,17]. However, it should take into account that this assumption is valid only for relative air humidity below 65% [18].

Another important issue is the possibility of surface resistance on boundary surfaces of wooden samples. This implies the adoption of more complex boundary conditions, e.g., boundary conditions of the third type instead of the first. This issue was considered experimentally by Rosen [19] analysing the effect of air velocity on water vapour adsorption for both longitudinal and transverse directions. He noted that for air velocities above 3 m/s, this effect is negligible and can be ignored.

The last issue, and probably the most important one, is the form of the source term. Its form has a very large impact on the results obtained from the model. A wrong one may give the apparent impression that, for example, the vapour diffusion coefficient is variable or that the transport of bound water is of great or minor importance.

The literature review shows that the topics related to moisture transport in the hygroscopic range are not fully explained and should be further analysed. This article proposes a model of moisture transport in wood that takes into account the diffusion and accompanying adsorption of water vapour through the skeleton. A new form of the source term has been proposed, which is an original contribution to modelling moisture flows in wood compared to those found in the literature. Its form was verified on thin pine samples for the relative humidity range from 30% to 50% and from 50% to 70%. The constancy of water vapour diffusion coefficients in the model for each of the orthotropic directions, boundary conditions of the first type for water vapour and boundary conditions with a source of moisture sorption for bound water were adopted. On the basis of measurements of changes in the mass of samples by minimising the target function, material coefficients were determined for the proposed model. The tests were performed on samples of the following dimensions 2 cm × 2 cm × 2 cm of pine, larch, oak and ash in the relative humidity range from 25% to 45%. The proposed model allowed to describe the process of moisture diffusion occurring in wood with a good fit for experimental research taking into account only three material parameters, i.e., the diffusion coefficient and two parameters describing the source term. Such a small number of model parameters allows to optimise the reverse methods of determining coefficients in wood and wood-based materials.

## 2. Model of Moisture Transport in Wood

### 2.1. Transport Equations

The moisture flow in wood in the hygroscopic range is mainly carried out by water vapour diffusion and sorption at the phase boundary between the air in the pores of the material and its skeleton. There is also a slight diffusion of bound water. However, it is much slower compared to the diffusion of water vapour in a porous structure [11] and thus negligible as mentioned in the introduction. For a one-dimensional case, without the influence of diffusion of bound water, the description of the phenomenon comes down to solving the following system of transport equations:

(1) $$\varepsilon \frac{\partial \rho_{v}}{\partial t}=D_{v}\frac{\partial^{2}\rho_{v}}{\partial x^{2}}-\dot{m},$$

(2) $$\rho_{w}^{dry}\frac{\partial C_{bw}}{\partial t}=\dot{m},$$

in which ε is the porosity of the material [m^3^/m^3^], $\rho_{v}$ is the water vapour density [kg/m^3^], $D_{v}$ is the water vapour diffusion coefficient in the material in one of the orthotropic directions [m^2^/s], $\dot{m}$ is the source term related to the moisture sorption process [kg/m^3^s], $C_{bw}$ is the moisture content (mass of water divided by mass of dry material) [kg/kg] and $\rho_{w}^{dry}$ is the density of dry wood [kg/m^3^].

For Equations (1) and (2) uniform initial conditions are adopted:

(3) $$\rho_{v}\left(x,t=0\right)=\rho_{v}^{0},$$

(4) $$C_{bw}\left(x,t=0\right)=C_{bw}^{0}\;$$

and boundary conditions of the first type for Equation (1):

(5) $$\rho_{v}\left(x=0,t\right)=\rho_{v}^{air},$$

(6) $$\rho_{v}\left(x=d,t\right)=\rho_{v}^{air},$$

where $\rho_{v}^{0}$ is the water vapour density corresponding to the initial relative air humidity in which the wood was conditioned [kg/m^3^], $C_{bw}^{0}$ is the moisture content corresponding to the initial relative air humidity in pores [kg/kg], $\rho_{v}^{air}$ is the water vapour density corresponding to the relative air humidity of the surrounding air [kg/m^3^], d is the sample thickness [m].

Such boundary conditions are correct for ambient air velocities greater than 3 m/s [18], which in the studies carried out in this paper was met (3.5–4 m/s).

For Equation (2), no boundary conditions are defined because the model does not assume bound water transport. Its changes are related only to the adsorption of water vapour, which takes into account the source term.

The change in average moisture content can be determined directly by measuring the mass of water that enters the material. It is also possible to determine the spatial distribution of the local moisture content in the sample. Isotopic or NMR methods are used [10,20]. The average moisture content is linked to the local moisture content by a relationship:

(7) $${\hat{C}}_{bw}\left(t\right)=\frac{1}{d}\int_{0}^{d}C_{bw}\left(x,t\right)\;dx.$$

### 2.2. Moisture Equilibrium

Moisture adsorption is the process responsible for the bounding of water vapour molecules on the inner surface of the material. The amount of absorbed moisture is greater the higher the relative air humidity and less the higher the temperature [21]. By studying the changes in the absorbed moisture with respect to relative air humidity, a so-called sorption isotherm is obtained, which describes how much the material is capable of absorbing water at a constant relative humidity and a constant temperature to move to a state of equilibrium. This equilibrium is determined by comparing the chemical potential of water vapour and bound water. For vapour, this potential is well defined on the basis of thermodynamic considerations and has the form (e.g., in [21]):

(8) $$\mu_{v}\left(T,\rho_{v}\right)=R_{v}\;T\;ln\left(\frac{\rho_{v}\;R_{v}\;T}{p_{v}^{sat}}\right),$$

in which $R_{v}$ is the gaseous constant of water [J/(kg K], T is temperature [K] and $p_{v}^{sat}$ is the saturation pressure of water vapour [Pa]. Several forms of this potential have already been proposed for water in porous materials [22,23,24], but it does not seem that any of them is particularly preferred. This article proposes a new form:

(9) $$\mu_{bw}\left(C_{bw}\right)=-\frac{1}{a}{\left(\frac{C_{bw}^{max}}{C_{bw}}-1\right)}^{\frac{1}{b}},$$

in which $C_{bw}^{max}$ is the maximum moisture content corresponding to fibre saturation point [kg/kg] while a and b are material constants expressed in [kg/J] and [-] respectively. By comparing Equations (8) and (9) and assuming that, for a state of equilibrium, the moisture content $C_{bw}$ becomes an equilibrium moisture content $C_{bw}^{eq}$ an expression is obtained for the moisture content at which the equilibrium between moisture balance between the material and the environment is achieved:

(10) $$C_{bw}^{eq}\left(T,\rho_{v}\right)=\frac{C_{bw}^{max}}{{\left(-a\;R_{v}\;T\;ln\left(\frac{\rho_{v}\;R_{v}\;T}{p_{v}^{sat}}\right)\right)}^{b}+1}$$

### 2.3. Source Term

As sorption stops at equilibrium, its rate should be a function of the difference between the equilibrium and the current moisture content:

(11) $$\dot{m}=k\;\left(C_{bw}^{eq}-C_{bw}\right)$$

where k is the coefficient describing the rate of adsorption [kg/(m^3^s].

However, the study [8] showed that the adsorption rate cannot be constant. This article adopts the original form of a source term similar to the function describing the chemical potential of associated water, with the difference that the maximum moisture content was replaced by an equilibrium moisture content:

(12) $$\dot{m}=k_{0}{\left(\frac{C_{bw}^{eq}}{C_{bw}}-1\right)}^{n}=k_{0}\frac{{\left(C_{bw}^{eq}-C_{bw}\right)}^{n-1}}{C_{bw}^{\;}{}^{n}}\left(C_{bw}^{eq}-C_{bw}\right)$$

where $k_{0}$ is the coefficient of absorption rate [kg/(m^3^s] and n is the material coefficient [-]. This form of function may be interpreted as depending on the difference between the equilibrium and the current moisture content with a variable adsorption coefficient or as depending on the distance of the current moisture content to the equilibrium state.

### 2.4. Water Vapour Diffusion Coefficient

The coefficient $D_{v}$ is the effective coefficient of water vapour diffusion, which may be related to the coefficient of water vapour diffusion in air according to the relationship:

(13) $$D_{v}=\frac{D_{va}}{\psi }$$

in which ψ is the dimensionless coefficient taking into account the resistance of the wood due to its porous structure and $D_{va}$ is the coefficient of water vapour diffusion in air. The coefficient $D_{va}$ adopted was as in [25] given as [26]:

(14) $$D_{va}\left(T\right)=2.16\cdot 10^{-5}\;\;{\left(\frac{T}{273.15}\right)}^{1.8}$$

## 3. Verification of the Adopted Forms of Sorption Isotherm and the Source Term

**Figure 1:**
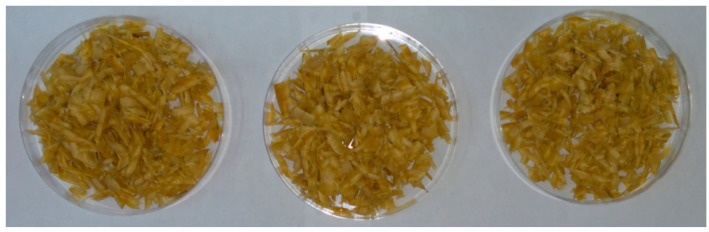
Thin wood samples used in the tests.

The verification of the functions describing the sorption isotherm and source term was carried out on wood chips obtained from pine wood with density 459.6 kg/m^3^ (Figure 1). They were about 0.1 mm thick. This allowed, in the case of the determination of the sorption curve, to significantly shorten the testing time in relation to the testing of samples of several centimetres and, in the case of the source term, to minimise the influence of diffusion on the adsorption process. This type of testing is also used for other fibrous materials [27].

Initially, the samples were dried at 60°C. After drying, they were moved to a climatic chamber with constant climatic conditions T=25°C and RH=30%. After the mass of the samples was stabilized (the mass changes of the samples were less than 0.002 g per 24 h), the relative humidity in the chamber was changed to RH=50%, and then the samples were weighed several times for 5 h. After the mass of the samples was stabilised again (about a week), the relative humidity in the chamber was changed again to RH=70% and the procedure was as in the previous stage. Once the mass was stabilised and the samples were weighed, they were placed in a exsiccator with relative air humidity RH≈98% and temperature T=25°C. This made it possible to determine the close to maximum moisture content that can be achieved by the wood still in the hygroscopic range.

On the basis of the measurements of the change of masses, the moisture content at sequent moments were calculated according to the formula:

(15) $$C_{bw}\left(t\right)=\frac{m_{w}\left(t\right)-m_{w}^{dry}}{m_{w}^{dry}}$$

where $m_{w}$ and $m_{w}^{dry}$ are the wet and dry masses of the sample. They were used to determine the parameters of the equation describing the sorption isotherm and the parameters of the model of moisture adsorption by wood.

**Figure 2:**
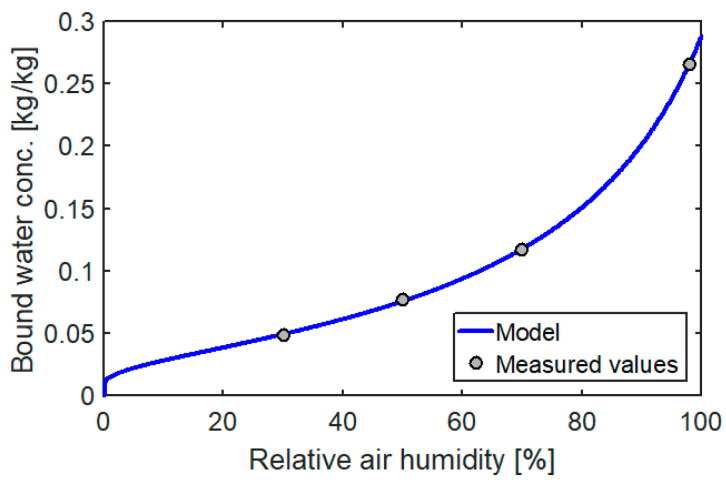
Sorption isotherm of the tested wood.

The coefficients of Equation (10) were determined by the Levenberg-Marquardt algorithm implemented in the Matlab package. The sorption isotherms for the determined parameters are shown in Figure 2.

A very good fit of the model curve to the data obtained from the measurements confirmed that Equation (9) describes the potential of bound water very well. In addition, it can be seen that in the range RH=20÷60% the dependence of the moisture content on relative air humidity is almost linear, which has been used in the calculation (shown in Section 6) to determine the equilibrium moisture content.

At this stage of research, a verification of the adopted source term was also carried out. For such thin samples, the water vapour density is almost immediately equalised in the entire pore volume. With a constant relative humidity of the surrounding air, we obtain, therefore, a homogeneous in the whole volume and constant in time distribution of the equilibrium moisture content $C_{bw}^{eq}=C_{bw}^{eq}\left(\rho_{v}^{air}\right)=const.$ This means that Equation (2), taking into account (12), is reduced to the form of:

(16) $$\frac{\partial C_{bw}}{\partial t}=h_{0}{\left(\frac{C_{bw}^{eq}=const.}{C_{bw}}-1\right)}^{n},\;h_{0}=\frac{k_{0}}{\rho_{w}^{dry}}$$

There is not analytical solution for the above equation. The solution was obtained using the finite difference method (FDM) with an explicit scheme. The coefficients of the equation were determined so that the values obtained according to Equation (16) were as close as possible to those obtained during the experiment. This was achieved by finding the minimum of the function F by means of a domain search. This function was in the form of:

(17) $$F\left(x\right)=\overset{N}{\underset{k=1}{\sum }}{\left(\frac{C_{bw,k}^{m,k}-C_{bw,k}^{c,k}}{C_{bw,k}^{m,k}}\right)}^{2}$$

where N is the number of measurements taken, $C_{bw,k}^{m}$ and $C_{bw,k}^{c}$ are the moisture contents measured and calculated according to the model, and x is the vector of sought parameters $h_{0}$ and n.

**Figure 3:**
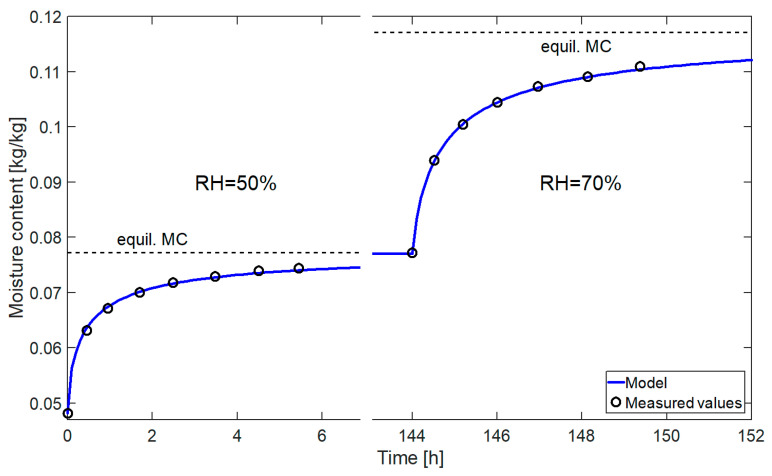
Change in moisture content in thin samples versus time.

The graph of the change of the moisture content versus time, obtained on the basis of the determined parameters, is shown in Figure 3.

In Figure 3 we can see a very good match between the values measured and obtained from the model in the humidity range from 30% to 70%. The material tested differed in terms of structure from the samples used in the main testing, but the results obtained confirm that the adopted form of the source term describes well the process of moisture adsorption by wood.

## 4. Description of Main Experiment

**Figure 4:**
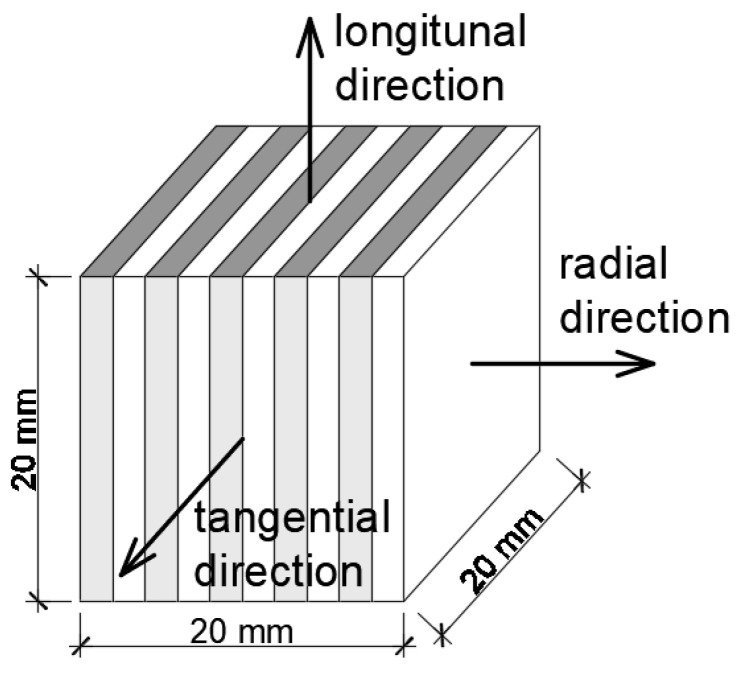
The cubic wood sample.

The main tests were performed on cubes with the following dimensions 2 cm × 2 cm × 2 cm, sourced from 4 types of wood (pine, larch, oak and ash), 36 pieces for each type. The samples were cut so that the growth rings are orthogonally aligned to their sides (Figure 4).

**Figure 5:**
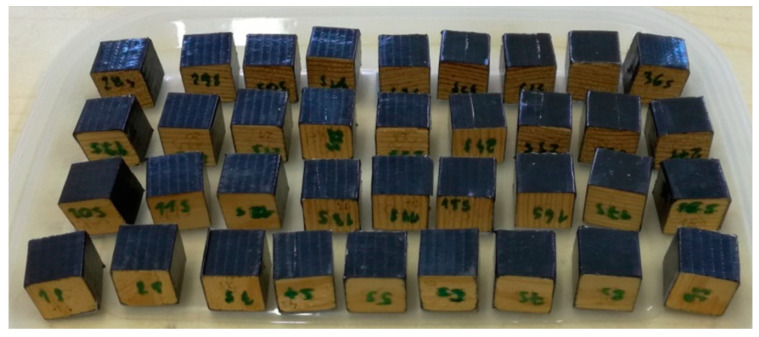
Insulated wood samples.

Initially, the samples were dried at 60 °C to determine the mass of dry material. They were then transferred to a climatic chamber with constant climatic conditions T=25 °C and RH=25%. Once the masses were stabilized (the mass changes of samples were less than 0.002 g per 7 days), the samples were insulated on four sides in such a way as to force a one-dimensional moisture flow. In this way 12 samples were obtained each of the three anatomical directions: radial, tangential and longitudinal (Figure 5) and weighed again to calculate the mass of insulation used.

The wooden cubes prepared in this way were placed in the climatic chamber where, after the masses were stabilised again, the air humidity was increased to RH=45%. The change in the mass of the samples on the first day was measured every several hours. The time between sequent successive measurements was gradually extended to 4 days in the final stage of measurements. The measurements of mass change took 39 days (total approx. 3 months). The measured masses were used to determine the average moisture content in the samples according to the formula:

(18) $${\hat{C}}_{bw}\left(t\right)=\frac{m_{w}\left(t\right)-m_{w}^{dry}}{m_{w}^{dry}}$$

where $m_{w}$ and $m_{w}^{dry}$ are the current mass and mass of dry sample respectively. Then, for each direction, 9 cubes were selected for which the results were the least divergent (only these were taken into account in the calculations). For these nine cubes the average concentration of the samples was calculated for each time moment according to the formula:

(19) $${\bar{C}}_{bw}=\frac{\sum_{i=1}^{P}{\hat{C}}_{bw}^{i}}{P}$$

where P=9 is the number of samples in the series.

For selected cubes the average apparent density of dry wood was calculated $\rho_{w}^{dry}$. The density of the wood substance for all types of wood is almost identical and depending on the research method [28] oscillate around 1500 kg/m^3^ (assumed as in [29] citing as in [30]). This allows to calculate the approximate porosity of the tested wood from the formula:

(20) $$\varepsilon =1-\frac{\rho_{w}^{dry}}{1500}\left[m^{3}/m^{3}\right]$$

The average densities and approximate porosities of the tested wood depending on its species are shown in Table 1.

The data obtained in this way made it possible to determine the coefficients of the model proposed in the article.

## 5. Numerical Solution

By discretising Equations (1) and (2), using the implicit and centred finite difference scheme, we obtained:

(21) $$\varepsilon \frac{{\left(\rho_{v}\right)}_{j+1}^{i}-{\left(\rho_{v}\right)}_{j}^{i}}{\Delta t}=D_{v}\frac{{\left(\rho_{v}\right)}_{j+1}^{i-1}-2{\left(\rho_{v}\right)}_{j+1}^{i}+{\left(\rho_{v}\right)}_{j+1}^{i+1}}{\Delta x^{2}}-{\dot{m}}_{j+1}^{i}$$

(22) $$\rho_{w}^{dry}\frac{{\left(C_{bw}\right)}_{j+1}^{i}-{\left(C_{bw}\right)}_{j}^{i}}{\Delta t}={\dot{m}}_{j+1}^{i}$$

where Δt is the time increase, Δx is the spatial increase, (j) is the index referring to the current moment in time for which the saturation values are known, (j+1) is the index referring to the future moment in time for which the saturation values are searching and (i−1, i) and (i+1) are the indices referring to spatial nodes.

In order to obtain a solution to Equations (21) and (22), it is more advantageous to present in a matrix form:

(23) $$A_{\alpha \beta }\left(y_{j+1}^{\;}\right)\frac{y_{j+1}^{\;}-y_{j}^{\;}}{\Delta t}+B_{\alpha \beta }\left(y_{j+1}^{\;}\right)\;y_{j+1}^{\;}=C_{\alpha }\left(y_{j+1}^{\;}\right)\;$$

where α, β=ρ, C, but $A_{\alpha \beta }$**,** $B_{\alpha \beta }$**,** $C_{\alpha }$ are matrixes of coefficients depending on state variables (in the matrix $B_{\alpha \beta }$ boundary conditions are taken into account), $y_{j}^{\;}=\left[{\left(\rho_{v}\right)}_{j}^{\;}\;{\left(C_{bw}\right)}_{j}^{\;}\right]$ is the vector of state variables in all spatial nodes for the (j)-th known point in time at which the initial conditions are taken into account, $y_{j+1}^{\;}$ is the saturation vector in all spatial nodes for the (j+1)-th search moment in time.

The value of the sought solution $y_{j+1}^{\;}$ is the argument $A_{\alpha \beta }$**,** $B_{\alpha \beta }$**,** $C_{\alpha }$. Therefore it is necessary to approximate the solution. This approximation was performed using the Newton-Raphson iterative method. The values of the variable in the (k+1)-th iteration in the (j+1)-th time step has a form [31]:

(24) $$y_{j+1}^{k+1}=y_{j+1}^{k}-{\left(\frac{\partial \Psi }{\partial y}\left(y_{j+1}^{k}\right)\right)}^{-1}\Psi \left(y_{j+1}^{k}\right)\;$$

where:

(25) $$\Psi \left(y_{j+1}^{k}\right)=A_{\alpha \beta }\left(y_{j+1}^{\;}\right)\frac{y_{j+1}^{\;}-y_{j}^{\;}}{\Delta t}+B_{\alpha \beta }\left(y_{j+1}^{\;}\right)\;y_{j+1}^{\;}-C_{\alpha }\left(y_{j+1}^{\;}\right)\;$$

The condition for the end of the calculation is in the form $∥\Psi \left(S_{j+1}^{k}\right)∥<\varepsilon$ where ε is a small value that determines the permissible error or assuming in advance the number of required iterations.

Based on the above algorithm, in the Matlab environment an original program solving the system of Equations (1)–(6) was written.

## 6. Optimization Procedure

The proposed model of moisture transport in wood has three parameters: two source term parameters $k_{0}$, n and the water vapour diffusion coefficient $D_{v}$. However, the tests lasted 39 days, which is too little time for the samples to reach equilibrium. The final moisture content $C_{bw}^{eq}$ (equilibrium moisture content) that the samples would achieve after a very long time was also required. So eventually, the number of unknowns rose to four parameters.

The model coefficients were determined in such a way that the differences between the moisture content determined during the experiment and calculated using the model were as small as possible. The target function was minimised by the domain method. In the case of a single series, the target function F was in the form of:

(26) $$F\left(x\right)=\overset{N}{\underset{i=1}{\sum }}{\left(\frac{{\bar{C}}_{bw}^{m,i}-{\bar{C}}_{bw}^{c,i}}{{\bar{C}}_{bw}^{m,i}}\right)}^{2}\;$$

where N is the number of measuring moments, ${\bar{C}}_{bw}^{m}$ and ${\bar{C}}_{bw}^{c}$ are the moisture contents measured and calculated according to the model in time $t_{i}$ and x is a vector for the sought parameters: $k_{0}$, n, $\psi =\frac{D_{va}}{D_{v}}$, $\Delta C_{bw}^{\infty }=C_{bw}^{eq}-C_{bw}^{last}$ ($C_{bw}^{last}$—last measured value).

Matching errors were calculated to evaluate the fitting of model results to the measured ones. Local error according to the formula:

(27) $$e_{l}\left(t_{i}\right)=\frac{\left|{\bar{C}}_{bw}^{m,i}-{\bar{C}}_{bw}^{c,i}\right|}{{\bar{C}}_{bw}^{m,i}}\;$$

and the global matching error according to the formula used in [32]:

(28) $$e_{g}=\frac{\sqrt{\sum_{i=1}^{N}{\left({\bar{C}}_{bw}^{m,i}-{\bar{C}}_{bw}^{c,i}\right)}^{2}}}{\sqrt{\sum_{i=1}^{N}{\left({\bar{C}}_{bw}^{m,i}\right)}^{2}}}\;$$

**Figure 6:**
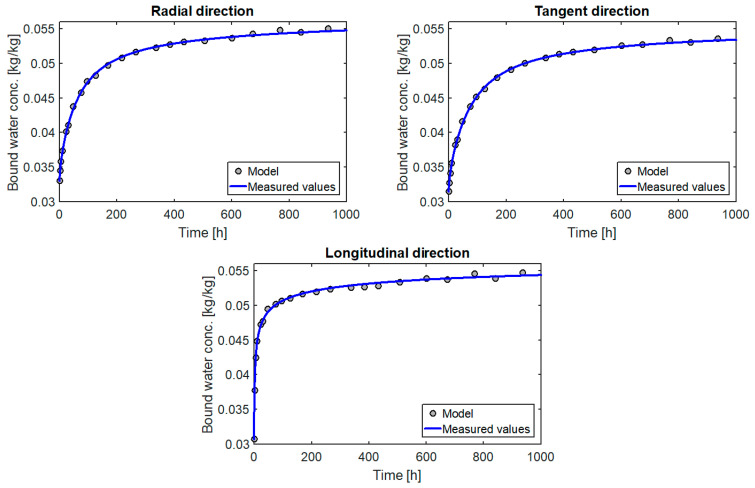
Diagrams of changes of moisture content for the pine samples.

The diagrams in Figure 6 show a very good fit of the model to the results obtained from measurements for all three directions of orthotropy. However, it can be seen that the coefficients of the source term shown in Table 2 differ slightly (although they should be the same because the source term is independent of direction). The reasons include measurement errors and heterogeneity of wooden samples. In order to determine the parameters k and n which would describe with a good approximation the moisture flow in all directions, the next step was to look for the best fit of the results for the three directions simultaneously.

**Figure 7:**
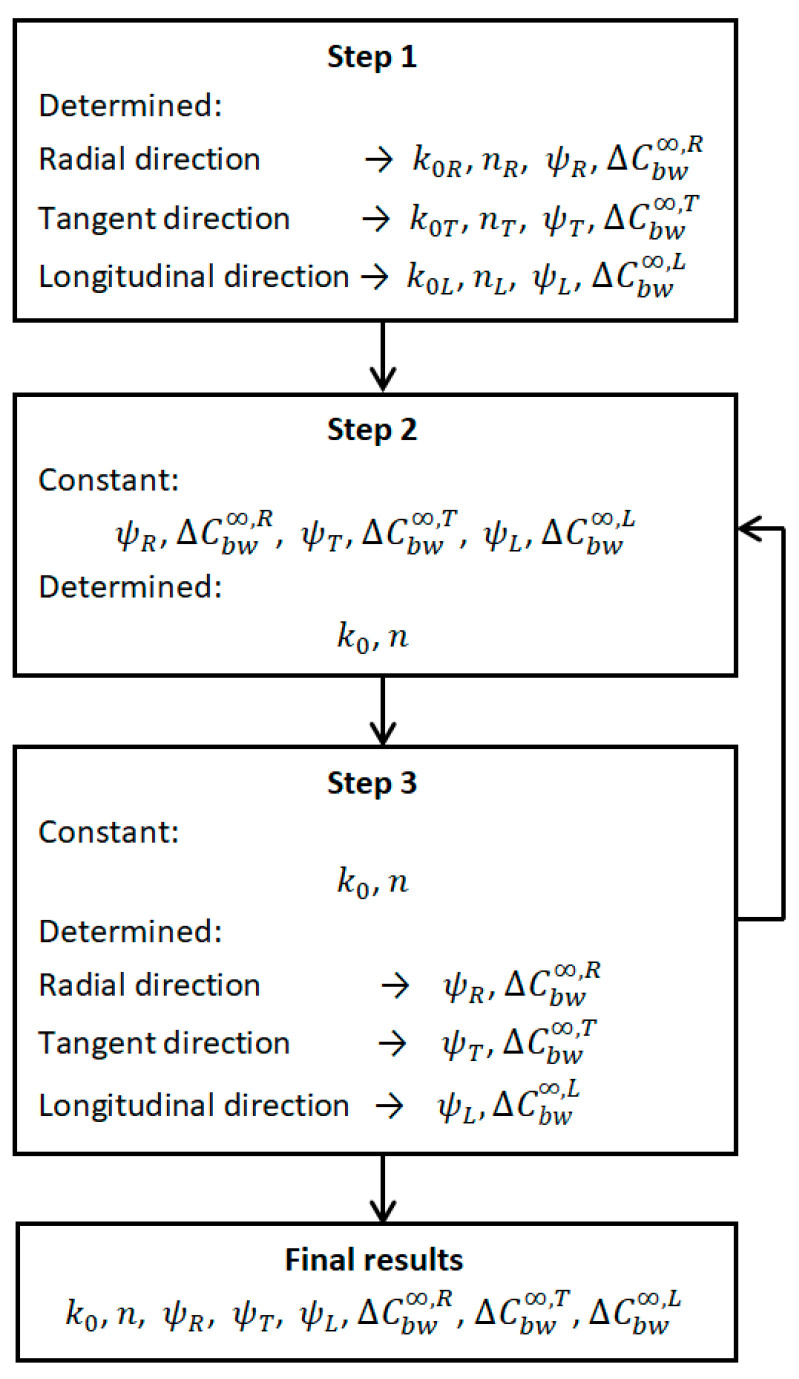
Algorithm for determining model coefficients.

Unfortunately in this case there are 8 coefficients to be determined. For this reason, this stage was divided into two parts. In the first one, for pre-determined diffusion coefficients and final moisture content, the parameters of the source term were determined by the domain method. In the second part, for the parameters set $k_{0}$ and n, the remaining coefficients for the best fit were sought. These calculations were repeated several times until the search values stopped changing. As shown in Table 3, for the results obtained in this way, the matching errors hardly changed compared to step one. This is due to the fact that the first stage already gives a very good approximation of the final results. The algorithm for determining coefficients of model is also shown in Figure 7.

In this way, calculations were made for all four sample types and the final results are presented in the next section.

## 7. Results and Discussion

As a result of calculations carried out in accordance with the scheme presented in Section 4, the parameter values of the proposed model of moisture transport in wood were obtained. They are presented in Table 4, Table 5, Table 6 and Table 7. For greater transparency and easier comparison of results, the diffusion coefficients are replaced by the vapour resistance factor for the successive orthotropic directions calculated according to the transformed formula (13):

(29) $$\psi_{R}=\frac{D_{va}}{D_{v,R}},\;\psi_{T}=\frac{D_{va}}{D_{v,T}},\;\psi_{L}=\frac{D_{va}}{D_{v,L}}$$

**Figure 8:**
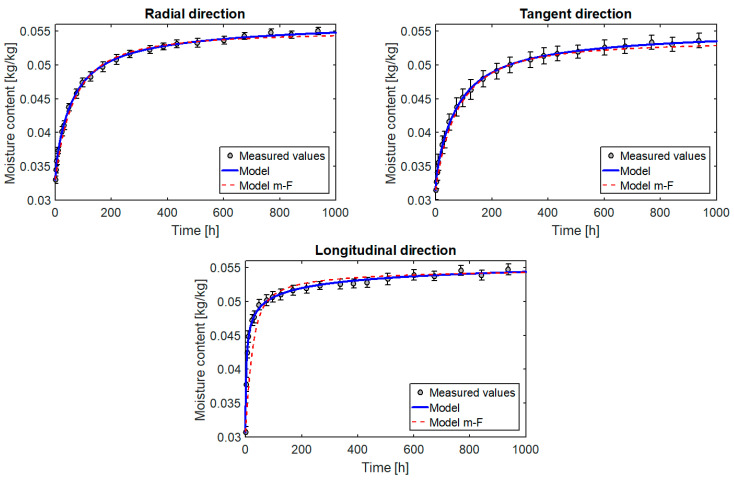
Changes in the moisture content of the pine samples versus time.

**Figure 9:**
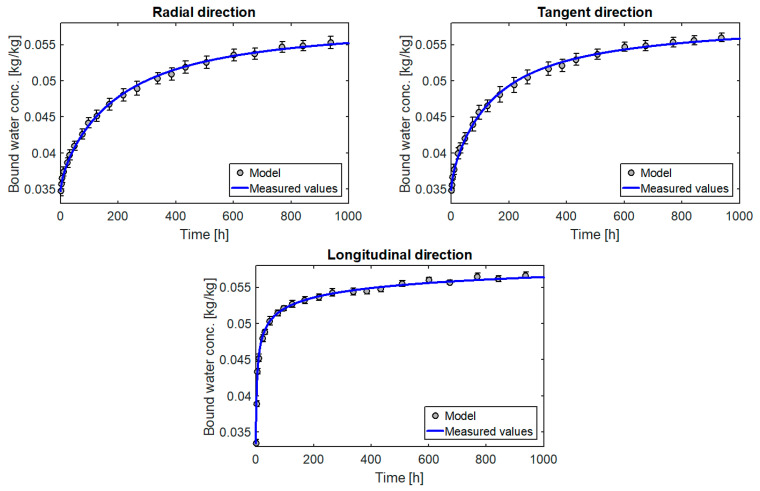
Changes in the moisture content of the larch samples versus time.

**Figure 10:**
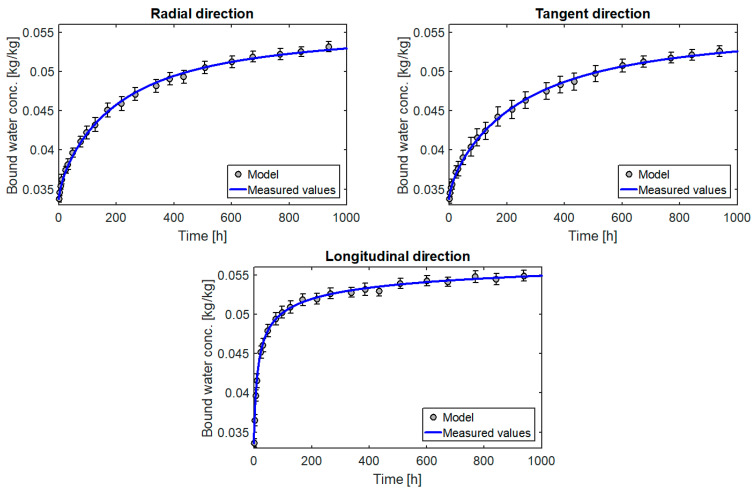
Changes in the moisture content of the oak samples versus time.

**Figure 11:**
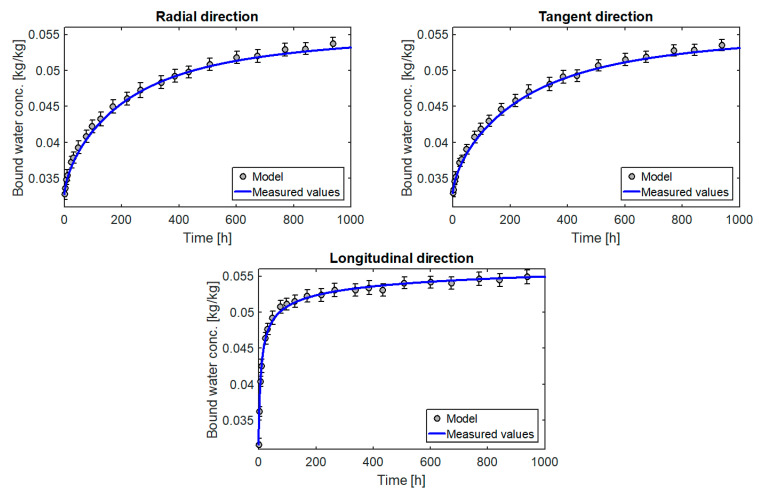
Changes in the moisture content of the ash samples versus time.

The results in graphical form are presented in Figure 8, Figure 9, Figure 10 and Figure 11. A model curve of the changes of moisture content ${\bar{C}}_{bw}$ versus time and its measured values together with the standard deviation was plotted. The obtained results were also compared with the results for the multi-Fickian model (model m-F) presented in [10] for parameters: $C_{1}=3.8\cdot 10^{-3}$ [1/s], $C_{3}=15$ [-], $C_{4}=5.94\cdot 10^{-7}$ [1/s], $C_{21}=3.58$ [-], $C_{22}=2.21$ [-],$C_{23}=1.59\cdot 10^{-3}$ [-], $C_{24}=14.98$ [-]. Standard deviation was calculated according to the formula:

(30) $$s=\sqrt{\frac{\sum_{i=1}^{P}{\left({\hat{C}}_{bw}^{i}-{\bar{C}}_{bw}\right)}^{2}}{P-1}}\;$$

where P is the number of samples in the series. Maximum and minimum values of standard deviation are presented in Table 8.

All the diagrams show a very good match between the curves obtained from the model and the measured values. This is confirmed by very small values of matching errors: local ($\max\left(e_{l}\right)=0.77\%÷1.71\%$) and global ($e_{g}=0.37\%÷0.97\%$). In addition, the model’s matching to the measurements is similar for the entire course of the test, which indicates that the model describes the process well for moisture levels far from and close to equilibrium.

The results of several researchers presented by Time [33] show that for spruce at RH=25% the values of diffusion resistance in the longitudinal and transverse directions should be in the ranges $\psi_{L}\in \langle 1.7;5.0\rangle$ and $\psi_{T}\in \langle 11;150\rangle$. Similarly Krabbenhoft and Damkilde [8] citing for Siau [13] show that $\psi_{L}\in \langle 1.7;2.8\rangle$ and $\psi_{T}\in \langle 56;156\rangle$, although for higher humidity. Diffusion resistances obtained from the model presented in Table 5, Table 6, Table 7 and Table 8 do not differ much from the values determined by these researchers.

The results of the calculations also confirm the correct selection of the form of the source term. The coefficients $k_{0}$ and n calculated for the three directions separately (Table 2) are very similar. A small difference is due to the heterogeneity of individual samples. In turn, the values calculated for the three directions simultaneously also describe the process very well for each direction. An incorrect selection of the form of the mass source would take effect of different values of parameters k and n for each direction or in the impossibility of obtaining, with constant diffusion coefficients, a satisfactory match between the calculation and the measurement.

For comparison, Figure 8 also shows the curves determined on the basis of the multi-Fickan model presented in [10]. This model also describes the process quite well (especially for the radial and tangential directions), but the source term in this model has as many as seven parameters, which makes it very difficult to use it for the reverse method. The form of the source term proposed here has only two parameters, but the fit to the experimental data is even better. All this confirms the suitability of the proposed model for modelling moisture transport in wood.

## 8. Conclusions

The model of moisture transport in wood proposed in the paper describes very well the diffusion process in the range of moisture present in the studies. This is confirmed by very small matching errors not exceeding for local 2% and for global 1%. The source term adopted in the model, although describing a very non-linear adsorption process and in a large humidity range, has only two parameters. The small number of unknowns makes it easy to determine the water vapour diffusion coefficients in wood from the reverse method. The main difference between this model and most of the literature is that the diffusion coefficients are constant and independent of the humidity of the wood and the surrounding air. However, the main tests were performed for a fairly narrow humidity range, and although tests performed on thin samples give a high probability of model accuracy for higher humidity ranges, it is necessary to extend the tests to a larger humidity range to allow more far-reaching conclusions to be drawn.

## Figures and Tables

**Table 1:** Average apparent densities and porosities of the dry wood used in the tests.

Type of Wood	Average Apparent Density of Dry Wood $\rho_{w}^{dry}\;[\mathrm{kg}/m^{3}]$	Porosity ε [m^3^/m^3^]
Pine	459.6	0.69
Larch	558.4	0.63
Oak	739.8	0.51
Ash	680.8	0.55

**Table 2:** Coefficients obtained for the pine samples.

Anatomical Direction	Diffusion Resistance Factor ψ[–]	Distance from Equilibrium Moisture Content $\Delta C_{bw}^{\infty }\;[\mathrm{kg}/\mathrm{kg}]$	Source Term Parameters	
			$k_{0}\cdot 10^{3}\;[\mathrm{kg}/(m^{3}s]$	n [–]
Radial R	68	0.0050	8.4	4.5
Tangential T	82	0.0048	6.4	4.3
Longitudinal L	2.1	0.0043	7.4	4.4

**Table 3:** Matching errors of the final results and of the first calculation stage for the pine samples.

Anatomical Direction	Matching Error [%]			
	Stage 1		Final Results	
	Maximum Local Error	Global Error	Maximum Local Error	Global Error
Radial R	0.86	0.42	0.89	0.43
Tangential T	1.0	0.37	0.99	0.38
Longitudinal L	1.36	0.62	1.46	0.63

**Table 4:** Model coefficients and matching errors for pine.

Anatomical Direction	Source Term Parameters		Vapour Resistance Factor ψ[-]		Error [%]	
	$k_{0}\cdot 10^{3}\;[\mathrm{kg}/(m^{3}s]$	n[-]			$\mathrm{Max}.\;\mathrm{Local}\;e_{l,max}$	$\mathrm{Global}\;e_{g}$
Radial R	6.9	4.4	$\psi_{R}$	65	0.86	0.42
Tangential T			$\psi_{T}$	83	1.0	0.37
Longitudinal L			$\psi_{L}$	2.0	1.46	0.63

**Table 5:** Model coefficients and matching errors for larch.

Anatomical Direction	Source Term Parameters		Vapour Resistance Factor ψ[-]		Error [%]	
	$k_{0}\cdot 10^{3}\;[\mathrm{kg}/(m^{3}s]$	n[-]			$\mathrm{Max}.\;\mathrm{Local}\;e_{l,max}$	$\mathrm{Global}\;e_{g}$
Radial R	8.2	5.4	$\psi_{R}$	157	1.38	0.58
Tangential T			$\psi_{T}$	114	1.13	0.59
Longitudinal L			$\psi_{L}$	2.4	0.90	0.49

**Table 6:** Model coefficients and matching errors for oak.

Anatomical Direction	Source Term Parameters		Vapour Resistance Factor ψ[-]		Error [%]	
	$k_{0}\cdot 10^{3}\;[\mathrm{kg}/(m^{3}s]$	n[-]			$\mathrm{Max}.\;\mathrm{Local}\;e_{l,max}$	$\mathrm{Global}\;e_{g}$
Radial R	7.0	4.5	$\psi_{R}$	135	1.58	0.62
Tangential T			$\psi_{T}$	165	0.77	0.42
Longitudinal L			$\psi_{L}$	6.8	1.10	0.44

**Table 7:** Model coefficients and fitting errors for ash.

Anatomical Direction	Source Term Parameters		Vapour Resistance Factor ψ[-]		Error [%]	
	$k_{0}\cdot 10^{3}\;[\mathrm{kg}/(m^{3}s]$	n[-]			$\mathrm{Max}.\;\mathrm{Local}\;e_{l,max}$	$\mathrm{Global}\;e_{g}$
Radial R	13.4	4.8	$\psi_{R}$	160	1.71	0.97
Tangential T			$\psi_{T}$	178	1.71	0.95
Longitudinal L			$\psi_{L}$	5.4	1.22	0.58

**Table 8:** Maximum and minimum values of standard deviation.

Type of Wood	Anatomical Direction	Standard Deviation [10^−2^ kg/kg]	
		Minimum	Maximum
Pine	Radial R	0.047	0.076
	Tangential T	0.097	0.146
	Longitudinal L	0.058	0.107
Larch	Radial R	0.061	0.096
	Tangential T	0.034	0.110
	Longitudinal L	0.030	0.059
Oak	Radial R	0.038	0.090
	Tangential T	0.052	0.123
	Longitudinal L	0.052	0.088
Ash	Radial R	0.069	0.106
	Tangential T	0.048	0.093
	Longitudinal L	0.066	0.097

## Data Availability

he data are included in this article (Figure 3, Figure 8, Figure 9, Figure 10 and Figure 11)
